# FPGA-Based Stochastic Echo State Networks for Time-Series Forecasting

**DOI:** 10.1155/2016/3917892

**Published:** 2015-12-31

**Authors:** Miquel L. Alomar, Vincent Canals, Nicolas Perez-Mora, Víctor Martínez-Moll, Josep L. Rosselló

**Affiliations:** Physics Department, University of the Balearic Islands, 07122 Palma de Mallorca, Spain

## Abstract

Hardware implementation of artificial neural networks (ANNs) allows exploiting the inherent parallelism of these systems. Nevertheless, they require a large amount of resources in terms of area and power dissipation. Recently, Reservoir Computing (RC) has arisen as a strategic technique to design recurrent neural networks (RNNs) with simple learning capabilities. In this work, we show a new approach to implement RC systems with digital gates. The proposed method is based on the use of probabilistic computing concepts to reduce the hardware required to implement different arithmetic operations. The result is the development of a highly functional system with low hardware resources. The presented methodology is applied to chaotic time-series forecasting.

## 1. Introduction

**Figure 1:**
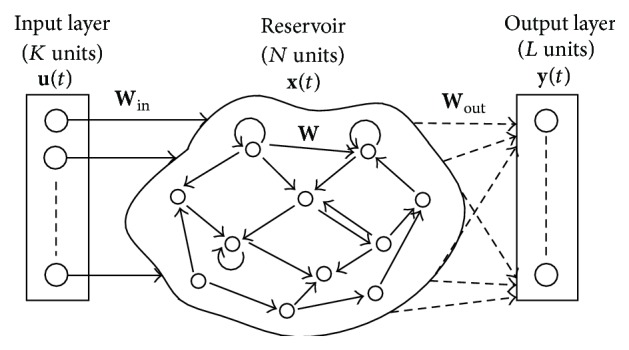
General architecture of RC systems. All connections in the reservoir are randomly chosen and kept fixed except for the ones that couple the reservoir on the output layer (dashed arrows).

*Introduction to Reservoir Computing*. Recurrent neural networks (RNNs) [1] are a class of artificial neural networks (ANNs) characterized by the existence of closed loops. RNNs are inspired by the way the brain processes information generating dynamic patterns of neuronal activity excited by input sensory signals [2]. Reservoir Computing (RC) [3–10] is a recently introduced efficient technique for implementing and configuring recurrent neural networks (RNNs). It is well suited for applications that require processing time dependent signals such as temporal pattern classification and time-series prediction [5]. In an RC system, all the interconnection weights of the RNN are kept fixed and only an output layer is configurable as illustrated in Figure 1. In recent years, RNNs have been extensively used to successfully solve computationally hard problems [11–15]. Nevertheless, the complex training procedure of RNNs is very time-consuming. On the other hand, RC presents an easy training procedure, which can be performed, in practice, via a simple linear regression.

The RC architecture is composed of three parts: an input layer, the reservoir, and an output layer (see Figure 1). The input layer feeds the input signals **u**(*t*) = (*u*_1_(*t*),…, *u*_*k*_(*t*)) to the reservoir with fixed random weight connections **W**_in_. The reservoir consists of a relatively large number of randomly interconnected neurons (*N*) with states **x**(*t*) = (*x*_1_(*t*),…, *x*_*N*_(*t*)) and internal weights **W**. Under the influence of input signals, the network exhibits transient responses which are read out at the output layer **y**(*n*) = (*y*_1_(*n*),…, *y*_*L*_(*n*)) by means of a linear weighted sum of the individual node states. As the only part of the system which is trained (assessment of the output weights **W**_out_) is the output layer, the training does not affect the dynamics of the reservoir itself unless a recurrence exists between the reservoir and the readout (recurrence weights given by **W**_back_).

The general expression to estimate the neuron states is given by

(1) $$\begin{array}{c} x\left(\;t+1\;\right)=f\left(\;W^{in}u\left(\;t+1\;\right)+Wx\left(\;t\;\right)+W^{back}y\left(\;t\;\right)\;\right), \end{array}$$

where **f** = (*f*_1_,…, *f*_*N*_) are the neuron transfer functions (typically sigmoidal). In the simplest case of avoiding direct connections between the input and the output layer as well as between the output and the reservoir, the output layer signals are computed according to the next expression:

(2) $$\begin{array}{c} y_{nn}\left(\;t+1\;\right)=\overset{N}{\underset{i=1}{\sum }}W_{i}^{out}\cdot x_{i}\left(\;t+1\;\right). \end{array}$$

**Figure 2:**
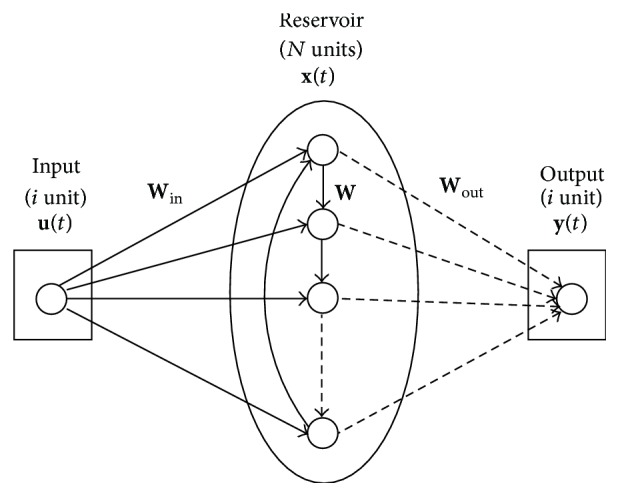
A simple cycle reservoir (SCR) topology. Units are organized in cycle.

A recent study [16] shows that a single reservoir ring is able to operate with similar efficiency to standard RC methodologies. The simplified topology is illustrated in Figure 2, where the connections between internal units in the reservoir have the same weight value *r*. The input layer is fully connected to the reservoir with a connection weight that will be positive or negative with equal probability and with the same absolute value for the weight (*v*). Parameters *r* and *v* must be scanned in order to find the optimum weight configuration.

It has been observed that the reservoir configured with a ring structure presents only a slightly worse performance than the classical topology [16]. Nevertheless, it presents some geometrical advantages that make it particularly useful for hardware implementation. The greatest benefit is that the number of connections within the reservoir is independent of the number of neurons *N* (while it increases with *N* in the case of the classical structure). This fact allows a great reduction of the number of required multipliers in the case of implementations using a high number of neurons. On the other hand, the design of the networks can be more easily automatized since all neurons have the same number of connections (one connection input from a neighboring neuron and a second connection from the input layer).

*Hardware Implementation of RC Systems*. Microprocessors are usually the preferred platform for implementing RC systems and, in general, ANNs. Nevertheless, there are specific applications that necessitate the use of compact, reliable, and energy efficient hardware realizations of ANN models [17]. Specialized ANN hardware can offer advantages over conventional PCs in terms of speed, power requirements, and cost. In addition, parallel hardware implementations offer substantial advantage in safety critical ANN applications where fault tolerance is crucial. FPGA is a suitable hardware for ANN implementation as it preserves the parallel architecture of the neurons in the network and offers flexibility in reconfiguration.

The RC principle can be used to implement computations on dynamical systems treating them as reservoirs. For example, it has been used to perform computation on hardware platforms such as analog electronics [18] and optoelectronic [19, 20] and optical systems [21]. Digital implementations of RC systems are limited to the use of spiking neurons (Liquid State Machine approach) [22]. To the best of our knowledge, the present paper is the first hardware implementation example of an RC system using classical sigmoid neurons (echo state network approach).

Despite the potential benefits of the hardware realization of ANNs, the implementation of massive neural networks in a single chip is a challenging task due to the fact that ANN algorithms are “multiplication-rich” and the multiplication operation is relatively expensive to implement [23].

Stochastic computing (SC) has evolved as a feasible alternative to implement complex computations due to the simplicity of the involved circuitry. It is based on the result of applying probabilistic laws to logic cells where variables are related to the random switching activity of internal bits [24, 25]. In general, stochastic logic considerably reduces the use of hardware resources if compared to traditional digital implementations. As an illustrative example, the product is performed by using a simple AND gate (see Figure 4). Several works have used the stochastic logic to implement feed forward neural networks [26–29]. In addition, there were some attempts to implement the RC framework using stochastic bitstream neurons [30, 31]. In general, a stochastic implementation is able to reduce the circuit area if compared to the classical one [32].

While the major benefits of SC are low hardware cost, low power requirements, and its inherent high error tolerance, the main drawback of SC is long computation time, which tends to grow exponentially with respect to precision. Over the years, SC has been recognized as potentially useful in specialized systems, where small size, low power, or soft-error tolerance is required and limited precision or speed is acceptable [33].

Even though SC-based ANNs seem unlikely to achieve speed-up compared to the conventional binary logic ones, they can be an interesting solution for those electronic systems implementing computational intelligence techniques and requiring low power dissipation but not demanding very high computational speed such as wireless sensor networks [34], predictive controllers, or medical monitoring applications. In the latter case, a software implementation of RC was found to achieve state-of-the-art performance in the classification of electrocardiographic signals [35]. Since this medical application requires a sampling time of about 1 ms, in general ECG classification would be compatible with an FPGA-based stochastic implementation of Reservoir Computing in real-time. Zhang and Li [36] present an illustrative example of the use of a SC-based ANN for the control unit of an induction motor exhibiting lower hardware cost than conventional microprocessor-based designs for the same application.

Another appealing feature of SC implementations is a high degree of error tolerance. Stochastic circuits tolerate environmental errors that seriously affect the behavior of conventional circuits. A single bit flip (especially of a high-significance bit) causes a huge error on a binary circuit, but flipping a few bits in a long bitstream has little effect in the value of the stochastic number represented. Therefore, SC can be interesting for applications like spacecraft electronics which operate under radiation-induced error conditions.

*Objectives and Contributions*. The aim of the present paper is to analyse the practicality of stochastic computing to build echo state networks (ESNs). We discuss the advantages and limitations of the proposed approach compared to a binary logic conventional implementation examining the hardware resource saving.

We specifically focus on the implementation of the reservoir system with ring topology of Figure 2 (SCR), which represents a considerable improvement considering the wiring optimization inside the integrated circuit. Results of the resource saving using the cyclic architecture compared with a standard ESN implementation presented in a preliminary study [37] are also reported.

Furthermore, we propose an implementation scheme for the stochastic ESNs which allows overcoming a major challenge of stochastic computing regarding the significant number of resources consumed by the stochastic number generators (SNGs) [38]. In particular, our design makes it possible to reduce the SNG count using common SNGs for all neurons. On the other hand, each neuron of the reservoir is constructed combining both stochastic and conventional binary logic to minimize the hardware area while maximizing the precision of computations (stochastic to implement the input weighting sum and classical for the activation function).

The proposed methodology is used to implement massive reservoir networks and applied to a challenging time-series prediction task.

## 2. Methods: Basic Concepts of Stochastic Computing

**Figure 3:**
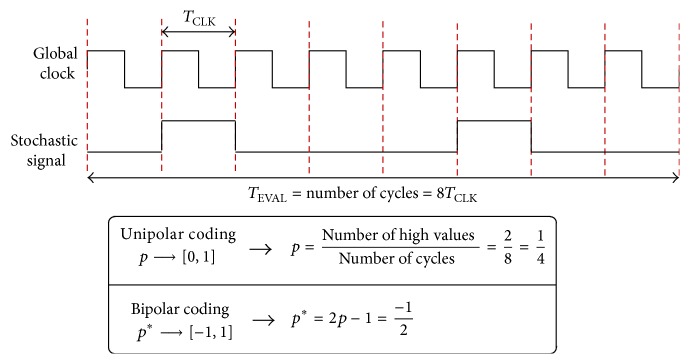
Basic concept of the stochastic codification. Information is coded as the probability “*p*” of the pulsed signal being in the high level.

In stochastic-based computations, a global clock signal provides the time interval during which all stochastic signals are stable (settled to 0 or 1, LOW or HIGH). During a clock cycle, each node of the circuit has a probability *p* of being in the HIGH state (see Figure 3). This probabilistic-based coding provides a natural way of operating with analog quantities (since probabilities are defined between 0 and 1) using digital circuitry.

### 2.1. Basic Arithmetic Circuitry

**Figure 4:**
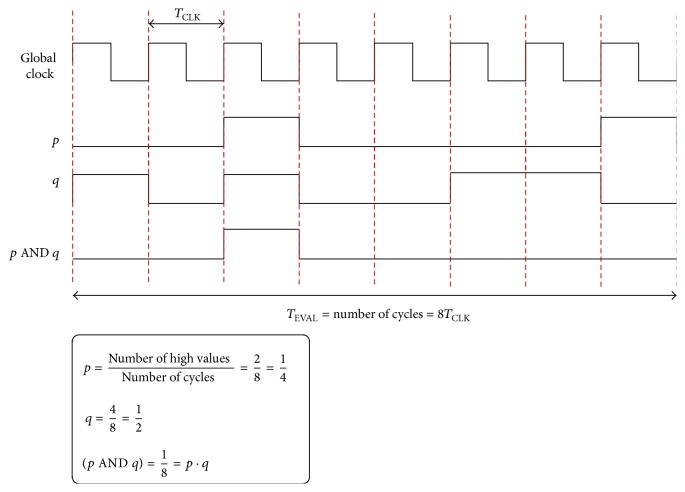
Product operation of two stochastic signals with switching activities *p* = 0.25 and *q* = 0.5 performed by means of an AND gate.

**Figure 5:**
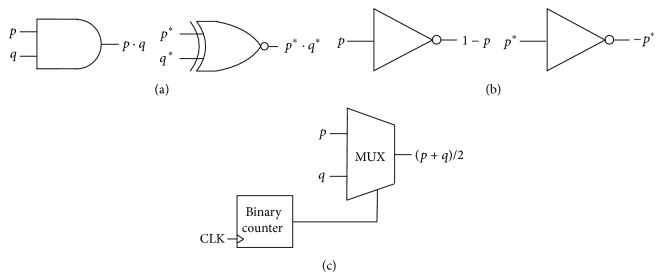
Stochastic arithmetic circuits. (a) Unipolar and bipolar multipliers. (b) Unipolar complementary operation and bipolar negation. (c) Adder used for both unipolar and bipolar notation.

Pulsed signals follow probabilistic laws when they are evaluated through logic gates. For instance, the AND gate provides at the output the product of their inputs (i.e., the collision probability between signals) as it is illustrated in Figure 4. Notice that the pulsed signals do not follow any particular pattern. Furthermore, they must be uncorrelated so that the operations can be performed properly. Some basic stochastic arithmetic circuits are depicted in Figure 5. A NOT gate converts the probability *p* at the input to the complementary 1 − *p* at the output. The mean value of two switching signals is implemented using a multiplexer and a binary counter. The counter supplies the selection signal to the multiplexer so that the output signal changes alternatively between *p* and *q*. One of the problems of this approach is that negative numbers cannot be represented directly since probabilities are positively defined. This inconvenience can be overcome by using a variable change with the switching probability *p* ^*∗*^ = 2*p* − 1 (this represents the bipolar coding in contrast to the unipolar coding used by just the probabilities). Therefore, since *p* is delimited between 0 and 1, *p* ^*∗*^ is bounded in the interval [−1, +1], and the zero value is located at *p* = 1/2. Using this notation the product is implemented by a single XNOR gate, the negation is obtained with a NOT gate, and the addition is implemented similarly to the unipolar codification (Figure 5).

### 2.2. Data Conversion

A stochastic computing system requires converting any real number (either in the unipolar [0,1] or in the bipolar [−1,1] range) represented by a binary magnitude *P* to its equivalent stochastic bitstream with probability *p* before the probabilistic computations. Similarly, the resulting pulsed signals must be finally converted into their equivalent binary values. The binary magnitude *P* representing the real number *P*_real_ is obtained by the simple formula *P* = [*P*_real_ · (2^*n*^ − 1)], where *n* is the number of bits employed to represent the real value. For example, the real value *P*_real_ = 0.5 is represented by the binary magnitude *P* = 32767 when using a 16-bit resolution. In the case of working with negative values (bipolar codification range), the numbers must be first normalized to the unipolar range. For instance, the real number *P*_real_^*∗*^ = −0.5 is represented by the value *P*_real_ = (*P*_real_^*∗*^ + 1)/2 = 0.25 in the unipolar range, which corresponds to the 16-bit binary magnitude *P* = 16383.

**Figure 6:**
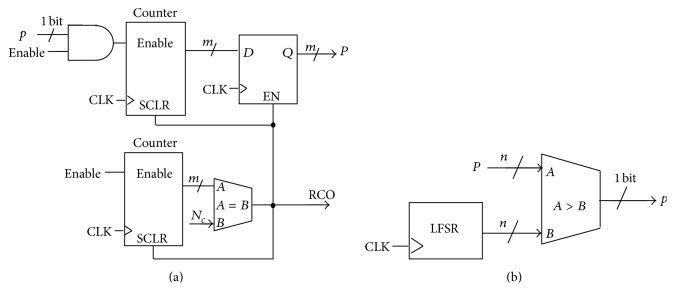
(a) Pulse to binary converter P2B(*N*_*c*_). (b) Binary to pulse converter B2P.

Binary numbers are converted to pulsed signals using a Binary to Pulsed (B2P) block; see Figure 6(b). This block is composed of a comparator and a linear feedback shift register (LFSR) used as pseudorandom number generator. Each clock cycle, the *n*-bit binary magnitude *P* is compared with a different random *n*-bit magnitude and the comparator provides a “1” if *P* is greater than the random magnitude and a “0” otherwise. Therefore, the output of the comparator provides a bitstream with probability (of getting a “1”) *p*. On the other hand, pulsed signals are converted to binary numbers using counters (Figure 6(a)). We define a pulse to binary converter of order *N*_*c*_ (a P2B(*N*_*c*_)) as the digital circuit that evaluates the number of HIGH values provided by a stochastic signal throughout *N*_*c*_ clock cycles. The output of a P2B(*N*_*c*_) block is an *n*-bit number that changes every *N*_*c*_ cycles so that the evaluation time is *T*_EVAL_ = *N*_*c*_ · *T*_CLK_ (where *T*_CLK_ is the clock cycle).

A probabilistic error is always present during conversions. When converting a switching signal with probability *p* by using a P2B(*N*_*c*_), the probability to obtain an output equal to *X* is given by the binomial distribution:

(3) $$\begin{array}{c} P\left(\;X\;\right)=\left(\begin{array}{c} N_{c}\\ X \end{array}\right)p^{X}{\left(\;1-p\;\right)}^{N_{c}-X}. \end{array}$$

The mean value of *X* is the expected exact conversion ($\overset{-}{X}=pN_{c}$), and the standard deviation at the output of the P2B circuit is $\sigma =\sqrt{N_{c}p\left(1-p\right)}$. This standard deviation is related to the error of the data conversion between the stochastic signal and the binary output of the P2B. This inherent error of the stochastic logic can be reduced by increasing the evaluation time although it decreases the processing speed of the system.

## 3. Methods: Proposed Stochastic Implementation of Neural Networks

**Figure 7:**
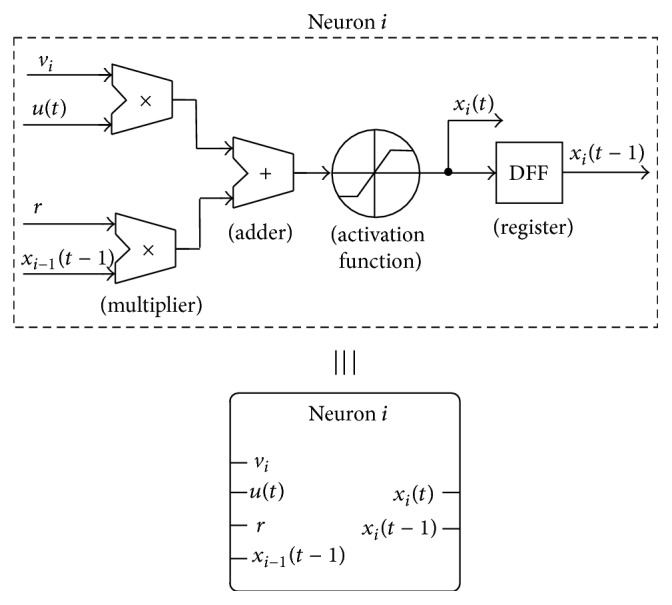
Operation and schematics of an artificial neuron with two inputs.

ESNs and, in general, ANNs are composed of individual artificial neurons performing a mathematical function. In particular, the neuron receives one or more inputs and sums them to produce an output. The sums of each node are weighted, and the sum is passed through a nonlinear function known as an activation function that usually has a sigmoid shape.  Figure 7 shows the operation and schematics of such a discrete-time artificial neuron with two inputs (here named *u*(*t*) and *x*_*i*−1_(*t* − 1)) weighted by their corresponding factors (*v*_*i*_ and *r*). Note that the output of the neuron is stored in a register so that it can be used by another neuron unit in the next time step in case the neurons are arranged in a recurrent network.

**Figure 8:**
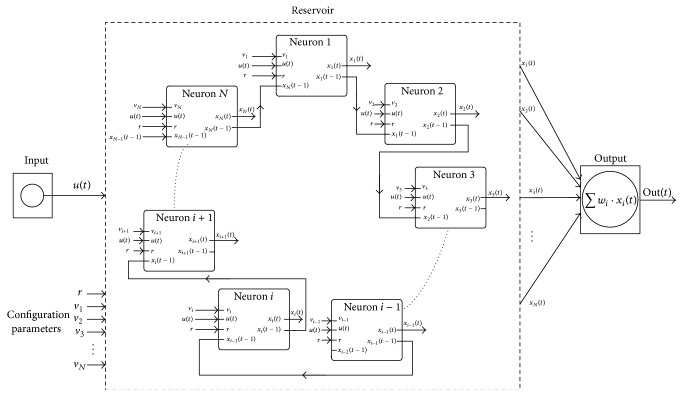
ESN with cyclic topology composed of two-input neurons.

Figure 8 shows how the individual neurons are organized to form an ESN with the cyclic architecture introduced in Figure 2.

**Figure 9:**
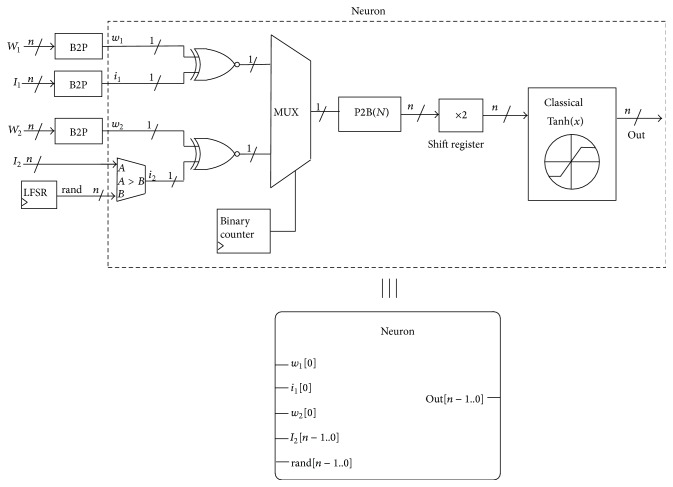
SC-based two-input sigmoid neuron. The linear part uses probabilistic logic whereas the nonlinear activation function is implemented classically. The output of the linear part is multiplied by 2 (shifting the binary word one position to left) to compensate the scaled sum performed by the multiplexer.

The SC-based implementation of the operations performed by the single neuron of Figure 7 is illustrated in Figure 9. First of all, the binary values of the inputs (external input and input coming from the output of another neuron) and their corresponding weights are transformed to pulsed signals (B2P blocks) so that they can be processed by the stochastic circuit. The multiplication and addition operations are implemented in the stochastic computing framework by means of an XOR gate and a multiplexer, respectively (when using the bipolar codification). Finally, the result of the input weighting and addition is evaluated by means of a sigmoid function.

**Figure 10:**
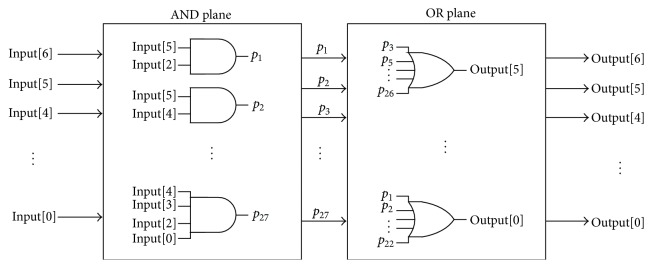
Implementation of the SIG-sigmoid function using direct bit-level mapping proposed by Tommiska [39].

Regarding the computation of the sigmoid function, which is a crucial issue for the neural implementation, there are different stochastic approaches to reproduce the hyperbolic tangent function [28, 29]. Nonetheless, for the present research, we have adopted a classical approximation of the hyperbolic tangent function called the SIG-sigmoid [39], which has proved to be an effective strategy in terms of accuracy, speed, and area resources. This classical approach consists of a purely combinational circuit that does not need arithmetic operators. A detailed description of this classical approximation is given in [39]. Basically, each one of the output bits of the sigmoid function is expressed in canonical form as a sum of products of the input bits. That is to say, the required input bits are firstly multiplied using AND gates, and then the resulting products are summed by a multiple-input OR gate. The SIG-sigmoid implementation concept using a direct bit-level mapping is illustrated in Figure 10.

**Figure 11:**
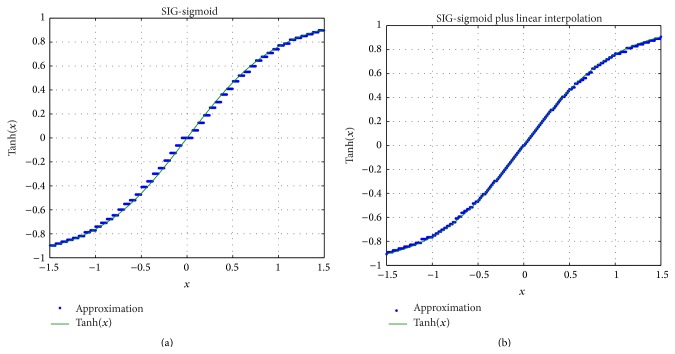
(a) Experimental measurements of the SIG-sigmoid function [39] and (b) of the approximation improved with linear interpolation.

Experimental measurements of the SIG-sigmoid approximation are presented in Figure 11(a). It can be noticed that this function exhibits discontinuities due to the fact that the precision of the implementation is limited to seven bits (for both input and output signals). We have overcome this limitation using a linear interpolation to obtain a greater number of bits for the output signal. The resulting function is displayed in Figure 11(b).

It can be appreciated in Figure 9 that the stochastic signal resulting from the weighting and addition of the inputs is converted (P2B block) to its corresponding binary value before it can be processed by means of the hyperbolic tangent classical approach. Therefore, we propose to combine the use of stochastic and conventional deterministic computing. Stochastic arithmetic allows reducing the computation hardware area required to implement the arithmetic operations present in neural networks while conventional binary logic can be used to increase the accuracy of the nonlinear activation function.

**Figure 12:**
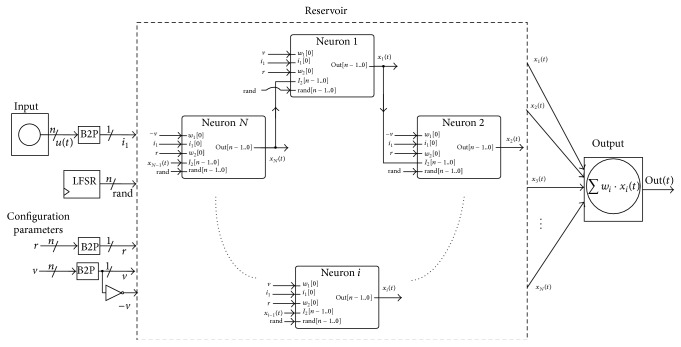
SC-based implementation of an ESN with cyclic architecture. A few pseudorandom number generators are shared by all neurons.

It is worth noticing that the four sequences of pseudorandom numbers required by the B2P converters contained in each neuron do not need to be different for each neuron since the neurons communicate with each other using binary magnitudes instead of probabilistic bitstreams (the output of a neuron is sent to another neuron as a binary value). The use of common random number generators for all the neurons allows reducing the number of required logic elements per neuron. Actually, the three B2P converter blocks (each containing a LFSR and an *n*-bit comparator) used for the weight parameters and first input value can be totally shared by all neurons since the weights and external input values are common for all neurons. The structure of the whole SC-based reservoir network using common B2Ps and LFSRs is depicted in Figure 12.

The greatest advantage of SC is the minimal use of resources for addition and multiplication. However, uncorrelated bitstreams and therefore large numbers of stochastic number generators (SNGs) are required. Since SNGs can account for a significant portion of the circuit [40], the reduction of the SNG count is an important challenge for SC. The NN implementation presented in this work enables using a few SNGs that are shared by all neuron units. This is possible since each individual neuron converts the output pulsed signal to a binary value before sending it to other neurons.

The hardware resource consumed by our SC-based implementation of ESNs is presented in next section for different network sizes. A breakdown indicating the logic elements used by each component of the neuron is also included.

Furthermore, a classical digital implementation of the ESN with cyclic architecture has been realized. The required operations illustrated in Figure 7 have been implemented using conventional binary logic. In particular, a 16-bit resolution has been used for the input and weight signals. A simple piecewise linear approximation of the hyperbolic tangent composed of three segments [41] has been used as activation function. This conventional hardware implementation serves as a reference to examine the hardware resource saving of the proposed stochastic approach.

A software program has been developed which allows the ESN structure (using either the SC approach or the deterministic one) to be exported automatically to a VHDL hardware description. The program generates the VHDL code for the reservoir with any desired number of neurons and weight configuration. This VHDL code can finally be synthesised to an actual hardware implementation.

## 4. Results

### 4.1. Proof-of-Concept Example

As an example of functionality of the proposed methodology, a small reservoir computer was synthesized on a Cyclone IV medium cost FPGA (see Table 1 for the spent hardware resources) and trained to perform different tasks. The reservoir is composed of 20 sigmoid neurons organized in a ring configuration as shown in Figure 2. No feedback connections are present between the output layer and the reservoir.

The output layer, which only requires a multiplier-adder circuit, was implemented using conventional binary logic with a resolution of 8 bits for the output weights. At the same time, a numerical model of the stochastic-based reservoir hardware is also developed with MATLAB for a more efficient training and debugging. The resolution of each variable is limited according to the hardware.

**Figure 13:**
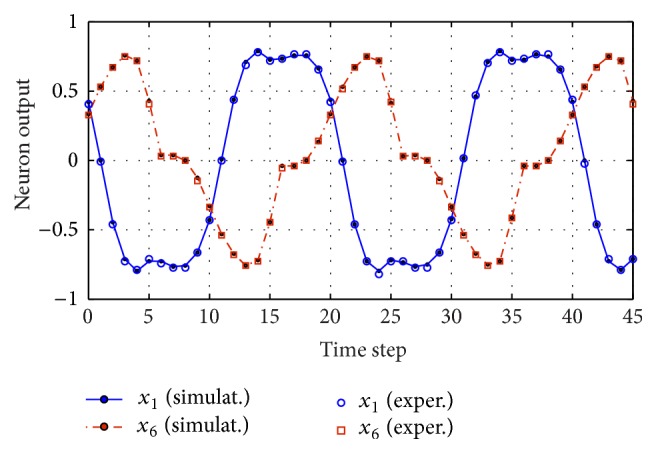
Traces of two arbitrarily selected neurons from the reservoir when driven by a sinusoid input. Experimental values (symbols) are plotted along with the numerical results (lines).

The first task selected to be performed is a nonlinear transformation of the input: *y*(*t*)_teach_ = (3/4)*u*(*t*)^3^. In particular, a single-channel sinusoid input *u*(*t*) = Sin⁡[2*π* · *t*/*T*] with 20 points per period (*T* = 20) was used to drive the system and supplied by an internal RAM memory to the reservoir every time step. This simple task will be useful to compare the FPGA implementation and the MATLAB model. In Figure 13, we show this comparison, where the dynamic of two randomly selected neurons from the reservoir is represented. As can be appreciated, a good agreement between the numerical simulations and the experimental results is obtained. Slight differences are mainly due to the probabilistic nature of the approach.

**Figure 14:**
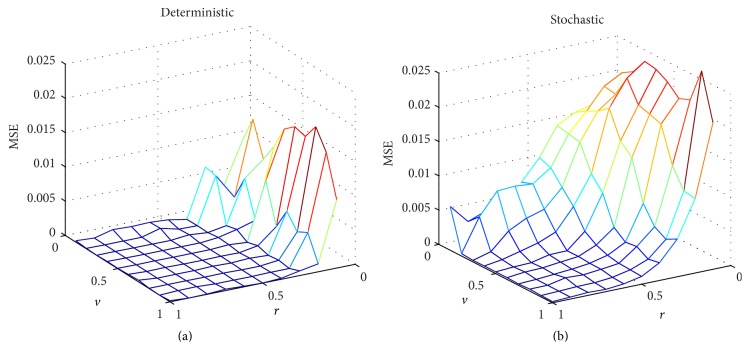
Simulation results for the mean square error (MSE) in the fitting task according to the classical and stochastic approaches. The two scanned parameters are *r* and *v*. The number of neurons was fixed to 20. A randomly generated distribution of the input weights was used. The evaluation time is fixed to *T*_EVAL_ = 2^16^ *T*_CLK_ for the stochastic approach.

The MATLAB model of the stochastic hardware allows us to perform a comprehensive assessment to find the configuration of the stochastic reservoir network providing optimum results. This is preferable to using a classical reservoir model since the optimum configuration values can be quite different between both scenarios. In Figure 14, we show the mean square error (MSE) for all the possible weight values of *v* and *r* in the reservoir. Furthermore, Figure 14 compares the MSE values for a classical 20-neuron reservoir (Figure 14(a)) and the MATLAB-simulated hardware implementation (Figure 14(b)).

Once the optimum parameters were determined, the hardware was configured, trained, and experimentally tested. The training (assessment of the output layer optimal weights) was carried out using the experimental outputs of individual neurons. This training consisted of a linear regression of the teacher output *y*_teach_ on the reservoir states. Although the software implementation quite faithfully reproduces the hardware results, it was not used to train the system since smaller error results were obtained when directly using the experimental outputs.

In the time-series experiment, we performed a total of 250 time steps. The first 20 time steps (the transient) are neglected, from *t* = 21 to *t* = 125 we trained the reservoir, and from *t* = 126 to *t*_max_ = 250 we tested the network.

**Figure 15:**
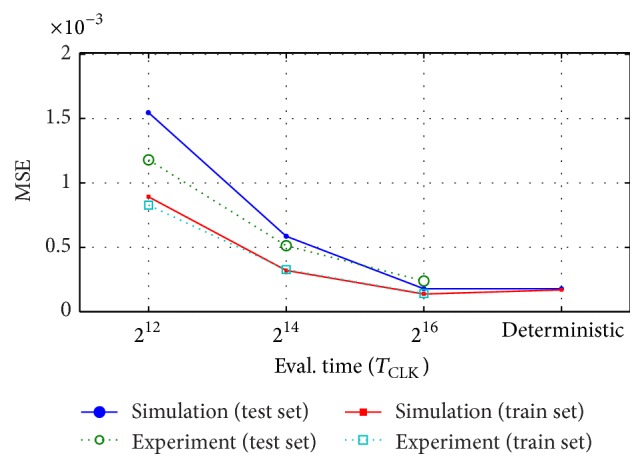
Simulation and experimental results of the mean square error (MSE) for the optimum reservoir configuration. The performance is represented for different evaluation periods. The estimated deterministic error is also given as a reference.

An experimental training error MSE_train_ = 1.41 · 10^−4^ and a test error of MSE_test_ = 2.39 · 10^−4^ were obtained when using a 16-bit precision. Other evaluation periods are represented in Figure 15, where we show both the MSE results for the MATLAB model of the FPGA implementation and the circuit measurements. The good agreement between simulations and experiments validates the MATLAB model to estimate the hardware performance. Furthermore, it can be observed that both stochastic reservoirs (MATLAB model and experimental measurements) gradually approach the expected deterministic behavior when increasing the evaluation time. The deterministic results were fixed with an 8-bit resolution for the neuron outputs and output weights.

**Figure 16:**
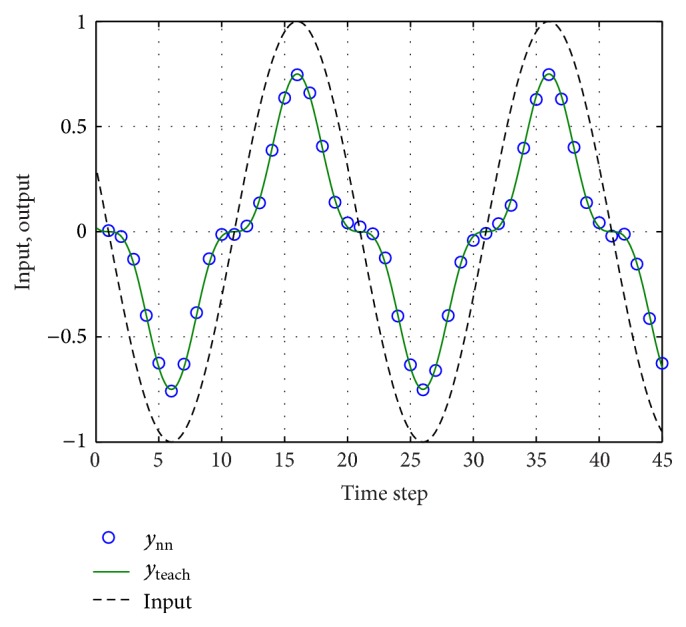
Input signal (Sin⁡[2*π* · *t*/20]) along with the desired output signal *y*_teach_ = (3/4)Sin^3^⁡[2*π* · *t*/20] and the experimental FPGA neural network output *y*_*nn*_ for the case of *T*_EVAL_ = 2^16^ *T*_CLK_.

Finally, we show in Figure 16 measurements at the output of the reservoir along with the expected behavior of the reservoir (*y*_teach_).

The evaluation time of the network is of the order of 1.3 ms when using 16-bit counters in the P2B(*N*) blocks (with a 50 MHz clock). The computation time can be reduced by using smaller evaluation periods at the cost of a lower accuracy. Note that, for larger reservoirs, the processing time is kept fixed since this is only dependent on the number of clock cycles used by the P2B converters.

### 4.2. Time-Series Prediction Task

A more complex task is implemented for a proper validation of the proposed methodology. This task consists in a one-step ahead prediction of the Santa Fe dataset [42]. The Santa Fe dataset is an experimental recording of the output power of a far-infrared laser when operating in a chaotic regime. We used 4000 samples from the original dataset; the first 2000 were used for training, the next 1000 for validation, and the remaining 1000 for testing.

**Figure 17:**
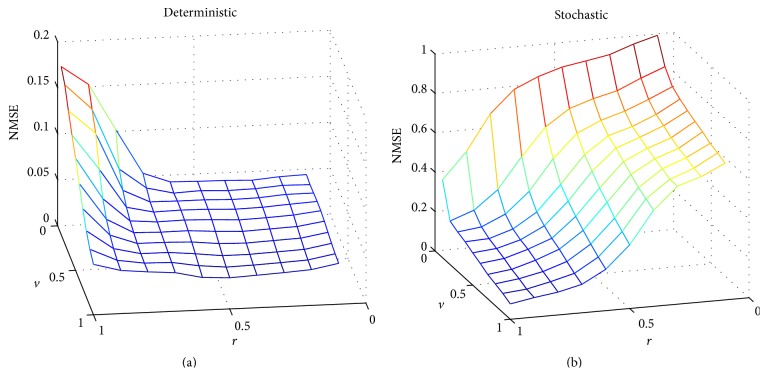
Simulation results for the normalized mean square error (NMSE) in the time-series prediction task according to the deterministic and stochastic approaches. The two scanned parameters are *r* and *v*. The number of neurons was fixed to 50. A randomly generated distribution of the input weights was used. The evaluation time is fixed to *T*_EVAL_ = 2^14^ *T*_CLK_ for the stochastic approach.

The analysis of this task was conducted using the MATLAB model of the stochastic hardware for two different reservoirs (with *N* = 20 and *N* = 50 neurons, resp.) and for different evaluation time periods. A procedure similar to the one used in the previous section is followed, first determining the optimum configuration and posteriorly testing the network.  Figure 17 shows the normalized mean square error (NMSE) as a function of the configuration parameters *r* and *v* for the 50-neuron reservoir and using a resolution of 14 bits. In Figure 17(a), we show the classical reservoir approximation and in Figure 17(b) we show the stochastic approach results (in both cases obtained using MATLAB simulations). As can be appreciated, the optimum values for *r* and *v* (that minimize the NMSE value) are different for both approaches. Therefore, the MATLAB model for the stochastic hardware is necessary for a proper estimation of the optimum weight values.

**Figure 18:**
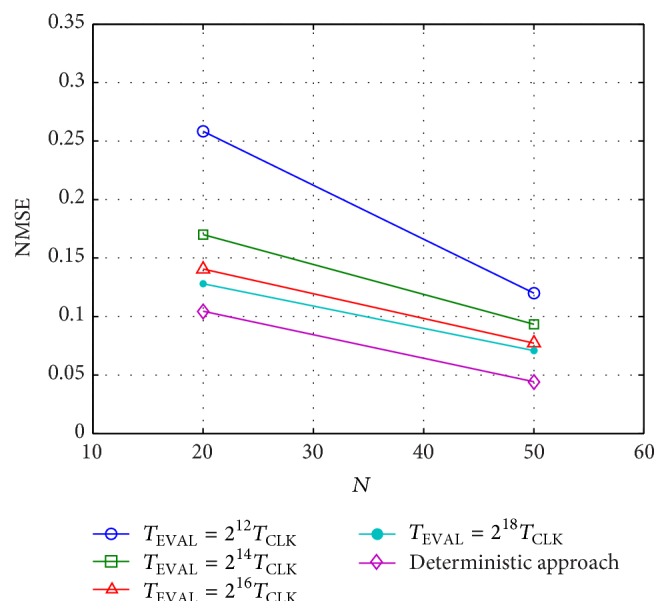
Normalized mean square error (NMSE) for the time-series prediction task. The stochastic-based results are displayed for a 20-unit and for a 50-unit reservoir using different values of the evaluation time. The corresponding results obtained with a deterministic approach are also represented.

The configuration parameters allowing the best performance error for the validation dataset were applied to the network when processing the test set. The final optimum results as a function of the number of neurons in the reservoir are depicted in Figure 18 with different bit precisions. It can be observed that the stochastic results gradually approach the deterministic ones when increasing the evaluation time.

**Figure 19:**
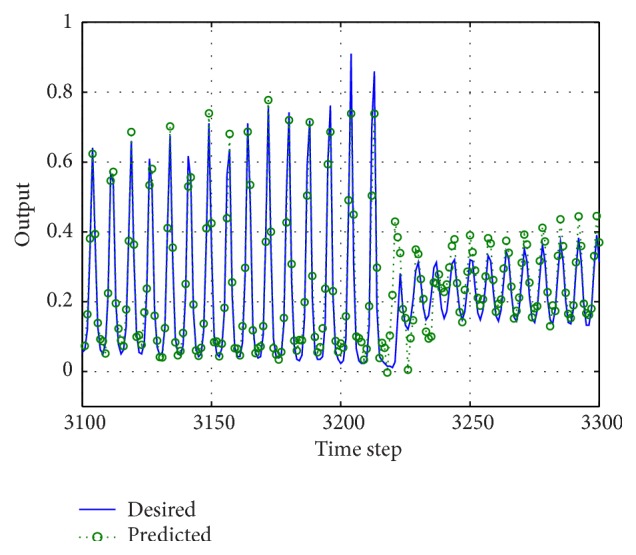
Segment of the laser time-series (predicted and targeted values). Predictions performed using the stochastic methodology with *N* = 50 and *T*_EVAL_ = 2^16^ *T*_CLK_.

In Figure 19, we show a fragment of the predicted and targeted laser intensity values for the stochastic reservoir when using *N* = 50 and a precision of 16 bits.

**Figure 20:**
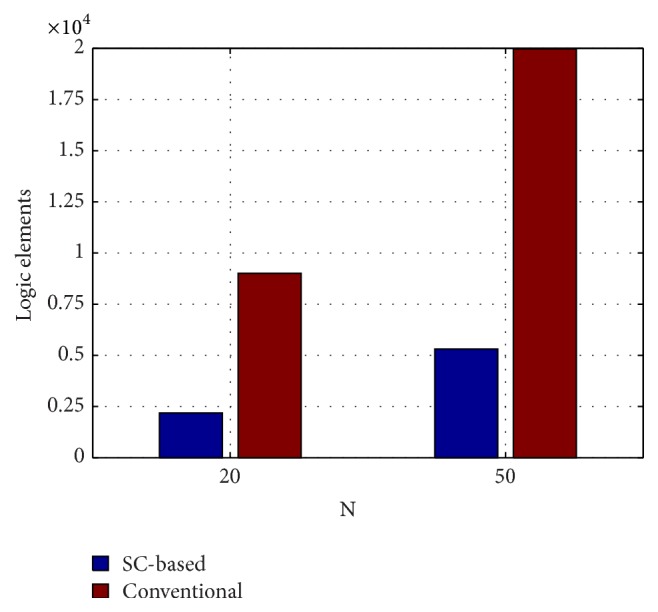
Comparison of the logic elements spent by the stochastic implementation and the conventional one.

The hardware resources required to implement the proposed stochastic-based reservoir networks together with the resources used for the conventional deterministic implementations are presented in Table 1 and in Figure 20. It can be observed that the stochastic architecture requires about four times less area than the conventional hardware implementation. It is worth noticing that the probabilistic methodology allows the massive implementation of reservoir networks in medium and even low cost FPGAs. However, the conventional implementation of the 50-neuron reservoir does not fit in low cost devices such as, for example, the Cyclone III EP316 containing about 15000 logic elements [43].

The implemented reservoir did not require any memory bits except the ones used to store the input data values. The values shown in Table 1 are referred only to the reservoir.

The use of the cyclic architecture allows significant resource saving compared to the standard random ESN implementation. A preliminary study [37], for instance, reported a requirement of about 2400 logic elements to build a 10-neuron ESN.

**Figure 21:**
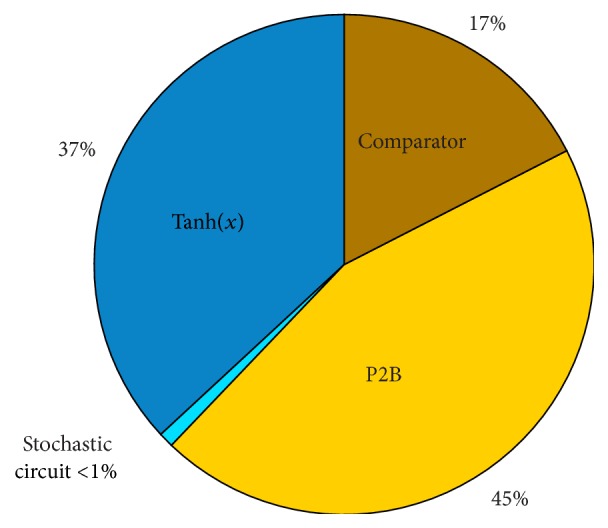
Breakdown of the hardware requirements of each component of the SC-based neuron.

A breakdown of the hardware requirements of each component of the SC-based neuron is illustrated in Figure 21. A typical stochastic neuron consumes 103 LEs of which only 1 is required by the stochastic circuit performing the additions and multiplications. The area cost of the architecture is dominated by the P2B converter (46 LEs) and by the hyperbolic function (38 LEs). Finally, the comparator uses 18 LEs.

The B2P converters, which are used as common elements by all the neurons, use 46 LEs each whereas the SNG (based on a 26-bit LFSR) consumes 26 LEs. Therefore, significant resource saving is achieved by sharing these components.

The proposed SC-based neuron design seems to be optimum in terms of hardware resources. Further reduction of the area requirements is only possible at the cost of a loss in accuracy using, for example, a rougher approximation of the sigmoid function or lower order P2B converters (the presented results are for neurons using pulsed signal conversions to 16-bit binary magnitudes).

## 5. Discussion

In this work, we have proposed and analysed an alternative architecture that exploits stochastic computing for doing time-series prediction with echo state networks. We have found that the stochastic architecture requires less area than a conventional hardware implementation. This characteristic makes the ESN implementation possible using low cost FPGA devices. Moreover, it has the advantage of being much more tolerant to soft errors (bit flips) than the deterministic implementation, which makes it particularly useful for applications that need to operate in harsh environments such as space.

However, it should be noted that the stochastic implementation requires relatively many clock cycles to achieve a given precision compared to a binary logic conventional implementation. For instance, to get a 16-bit resolution, a computation time of 2^16^ clock cycles is needed.

Therefore, in general, potential applications of the stochastic implementations are specialized systems where small size, low cost, low power, or soft-error tolerance is required, and limited speed is acceptable. The presented SC-based ESN approach can be an interesting solution, by way of example, for electronic systems implementing computational intelligence techniques and requiring low power dissipation such as wireless sensor networks, predictive controllers, or medical monitoring applications.

For the ESN, a ring topology has been selected since hardware resources are minimized with this configuration while the precision of the network is not decreased with respect to a classical random one. In addition, we have proposed an implementation area-efficient scheme that employs probabilistic logic for the arithmetic operations and conventional binary logic for the nonlinear activation function. This scheme allows reducing the number of SNGs, which are expensive in terms of hardware resources, using common SNGs for all neurons. It has been observed that the area cost of the proposed implementation is dominated by the P2B converters and the sigmoid function.

The proposed methodology has been used to implement a massive reservoir network and has exhibited considerable performance in a chaotic time-series prediction task.

Reservoir networks present some advantages compared to conventional recurrent neural networks that enable a more efficient hardware implementation. A major benefit of RC networks is their sparse connectivity. This characteristic allows a simple wiring that matches the FPGA capabilities. Additionally, a simple training process can be performed offline.

The use of the stochastic logic implies certain constraints. The shortcomings are the evaluation time and the precision. Nevertheless, these drawbacks are compensated for by the much simpler architecture and by the stochastic logic's inherent noise immunity which, all in all, allow a massive, parallel, and reliable implementation.

## Figures and Tables

**Table 1:** Spent hardware resources of the medium-sized Cyclone IV (EP4CE115F297C7N) FPGA for the 20-unit and 50-unit reservoir networks.

Implementation approach	Stochastic		Conventional	
Reservoir size	20 neurons	50 neurons	20 neurons	50 neurons
Total logic elements (LEs)	2186 (1.9%)	5306 (4.6%)	9013 (7.9%)	19975 (17.4%)
Combinational functions	2149 (1.9%)	5251 (4.6%)	9013 (7.9%)	19975 (17.4%)
Dedicated logic registers	858 (0.7%)	2054 (1.8%)	320 (0.3%)	800 (0.7%)
